# Author Correction: WDR77 inhibits prion-like aggregation of MAVS to limit antiviral innate immune response

**DOI:** 10.1038/s41467-023-41712-w

**Published:** 2023-09-25

**Authors:** Jiaxin Li, Rui Zhang, Changwan Wang, Junyan Zhu, Miao Ren, Yingbo Jiang, Xianteng Hou, Yangting Du, Qing Wu, Shishi Qi, Lin Li, She Chen, Hui Yang, Fajian Hou

**Affiliations:** 1 State Key Laboratory of Molecular Biology, Shanghai Institute of Biochemistry and Cell Biology, Center for Excellence in Molecular Cell Science, Chinese Academy of Sciences; University of Chinese Academy of Sciences, Shanghai, 200031 China; 2 Key Laboratory of Systems Health Science of Zhejiang Province, School of Life Science, Hangzhou Institute for Advanced Study, University of Chinese Academy of Sciences, Hangzhou, 310024 China; 3 National Institute of Biological Sciences, Beijing, 102206 China; 4 Shanghai Key Laboratory of Brain Function Restoration and Neural Regeneration, Huashan Hospital, Shanghai Medical College, Fudan University, Shanghai, 200032 China

**Keywords:** RIG-I-like receptors, Molecular biology

Correction to: *Nature Communications* 10.1038/s41467-023-40567-5, published online 10 August 2023

The original version of this Article contained errors in the legends of Figures 1–4, 6, and 7.

The legend of Figure 1 originally incorrectly read ‘*P*-value was determined by two-tailed Student’s *t*-test.’ The correct version replaces this sentence with ‘*P*-values were determined by two-way ANOVA (Dunnett’s test) (**f**) or two-way ANOVA (Šídák’s test) (**g**, **h**, **i**, **j** and **k**).’

The legend of Figure 2 originally incorrectly read ‘*P*-value was determined by two-tailed Student’s *t*-test.’ The correct version replaces this sentence with ‘*P*-values were determined by two-way ANOVA (Šídák’s test) (**a**, **b**, **d**–**f**, **i**, **j**), ordinary one-way ANOVA (Tukey’s test) (**h**) or two-way ANOVA (Tukey’s test) (**l**).’

The legend of Figure 2h originally incorrectly read ‘GFP-VSV titers were quantitated in plaque assay (For **d**, VSV: *p = 0.0123, ****p < 0.0001 in sequence).’ The correct version states ‘For **h**’ instead of ‘For **d**’.

The legend of Figure 2l originally incorrectly read ‘*IFNB1* induction was measured by qPCR (For **l**, *IFNB1*: ****p < 0.0001, ***p = 0.0001, ^ns^*p* = 0.9957 in sequence).’ The correct version states ‘***p = 0.0004’ instead of ‘***p = 0.0001’.

The legend of Figure 3 originally incorrectly read ‘*P*-value was determined by two-tailed Student’s *t*-test.’ The correct version replaces this sentence with ‘*P*-values were determined by two-way ANOVA (Šídák’s test) (**a**–**c**, **h**) or unpaired two-sided *t*-test (**e**).’

The legend of Figure 3h originally incorrectly read ‘*IFNB1* induction was measured by qPCR (*ISRE*: all ****p < 0.0001; ^ns^*p* = 0.9998).’ The correct version states ‘*IFNB1*’ instead of ‘*ISRE*’.

The legend of Figure 4 originally incorrectly read ‘*P*-value was determined by two-tailed Student’s *t*-test.’ The correct version replaces this sentence with ‘*P*-values were determined by two-way ANOVA (Šídák’s test) (**i**).’

The legend of Figure 4i originally incorrectly read ‘Cells were harvested at specified time points post infection and *IFNB1* induction was measured by qPCR (*IFNB1*: all ^ns^*p* > 0.9999, ***p < 0.0001, **p = 0.0018).’ The correct version states ‘****p < 0.0001’ instead of ‘***p < 0.0001’.

The legend of Figure 6 originally incorrectly read ‘*P*-value was determined by two-tailed Student’s *t*-test.’ The correct version replaces this sentence with ‘*P*-values were determined by two-way ANOVA (Tukey’s test) (**a**, **b**, **g**), two-way ANOVA (Šídák’s test) (**c**) or two-way ANOVA (Dunnett’s test) (**d**–**f**).’

The legend of Figure 7 originally incorrectly read ‘*P*-value was determined by two-tailed Student’s *t*-test.’ The correct version replaces this sentence with ‘*P*-values were determined by two-way ANOVA (Tukey’s test) (**a**, **c**), two-way ANOVA (Šídák’s test) (**b**, **d**, **h**) or Gehan-Breslow-Wilcoxon test (**f**, **g**, **i**).’

The legend of Figure 7i originally incorrectly read ‘WT, *Wdr77*^CKO^ and *Mavs*^-/-^ mice (*n* = 10 each) were injected intravenously with HSV at 1.5 × 10^8^ PFU per mouse, and the survival rates were monitored for 15 days (*p = 0.445).’ The correct version states ‘^ns^*p* = 0.445’ instead of ‘*p = 0.445’.

This has been corrected in both the PDF and HTML versions of the Article.

The original version of this Article contained errors in the legends of supplementary Figures 1–5, 7, and 8.

The legend of supplementary Figure 1 originally incorrectly read ‘*P* value was determined by two-tailed Student’s *t*-test.’ The correct version replaces this sentence with ‘*P* values were determined by two-way ANOVA (Dunnett’s test) (**a**, **b**).’

The legend of supplementary Figure 2 originally incorrectly read ‘*P* value was determined by two-tailed Student’s *t*-test.’ The correct version replaces this sentence with ‘*P* values were determined by unpaired two-sided *t*-test (**b**), ordinary one-way ANOVA (Dunnett’s test) (**e**) or two-way ANOVA (Šídák’s test) (**c**, **f**, **g**).’

The legend of supplementary Figure 3 originally incorrectly read ‘*P* value was determined by two-tailed Student’s *t*-test.’ The correct version replaces this sentence with ‘*P* values were determined by two-way ANOVA (Šídák’s test) (**b**–**e**, **g**, **i**).’

The legend of supplementary Figure 4 originally incorrectly read ‘*P* value was determined by two-tailed Student’s *t*-test.’ The correct version replaces this sentence with ‘*P* values were determined by two-way ANOVA (Šídák’s test) (**c**, **e**, **f**, **h**).’

The legend of supplementary Figure 5 originally incorrectly read ‘*P* value was determined by two-tailed Student’s *t*-test.’ The correct version replaces this sentence with ‘*P* values were determined by two-way ANOVA (Šídák’s test) (**g**).’

The legend of supplementary Figure 7 originally incorrectly read ‘*P* value was determined by two-tailed Student’s *t*-test.’ The correct version replaces this sentence with ‘*P* values were determined by ordinary one-way ANOVA (Dunnett’s test) (**e**, **f**), two-way ANOVA (Dunnett’s test) (**g**, **i**–**k**) or two-way ANOVA (Šídák’s test) (**h**).’

The legend of supplementary Figure 8 originally incorrectly read ‘*P* value was determined by two-tailed Student’s *t*-test.’ The correct version replaces this sentence with *‘P* values were determined by two-way ANOVA (Dunnett’s test) (**a**, **c**, **d** (liver), **e** (lung), **f** (spleen, lung)), two-way ANOVA (Tukey’s test) (**b**, **d** (spleen, lung), **e** (spleen), **f** (liver)), or two-way ANOVA (Šídák’s test) (**e** (liver)).’

The HTML has been updated to include a corrected version of the Supplementary Information.

The original version of this Article contained errors in the Statistical Analysis in the Methods.

The Statistical Analysis in the Methods originally incorrectly read ‘Unpaired two-sided Student’s *t*-test was used to analyze the significance of mean values between groups. Kaplan–Meier survival analysis was used to analyze mouse survival data by two-sided log-rank (Mantel–Cox) test.’

The correct version replaces these sentences with ‘Unpaired two-sided Student’s *t*-test, ordinary one-way ANOVA and two-way ANOVA were used to analyze the significance of the differences between groups. Survival data of mice were analyzed using Gehan-Breslow-Wilcoxon test.’

This has been corrected in both the PDF and HTML versions of the Article.

The original version of this Article contained an error in the Source Data for Figure 2h.

The Source Data for Figure 2h originally incorrectly read ’35.6, 27.2 and 24.8’ for VSV titer in WT, and ‘7.4, 6 and 8’ for VSV titer in *WDR77*^-/-^ and omitted measurements for *MAVS*^-/-^. The correct version replaces these values with ‘410000000, 310000000 and 370000000’ for VSV titer in WT, and ‘44000000, 38000000 and 34000000’ for VSV titer in *WDR77*^-/-^, and adds ‘1200000000, 1500000000 and 1300000000’ for VSV titer in *MAVS*^-/-^.

The HTML has been updated to include a corrected version of the Source Data.

### Supplementary information

Updated Supplementary Information

Updated Source Data

