# Supplementary material for: WDR77 inhibits prion-like aggregation of MAVS to limit antiviral innate immune response
Source: Nat Commun. 2023 Aug 10;14:4824. doi: 10.1038/s41467-023-40567-5 (PMC10415273; doi:10.1038/s41467-023-40567-5)

Supplementary Information

WDR77 inhibits prion-like aggregation of MAVS to limit antiviral innate immune response

Jiaxin Li[1], Rui Zhang[1], Changwan Wang[1, 2], Junyan Zhu[1], Miao Ren[1], Yingbo Jiang[1], Xianteng Hou[1], Yangting Du[1], Qing Wu[1], Shishi Qi[1], Lin Li[3], She Chen[3], Hui Yang[4], Fajian Hou[1, 2, *]

[1]State Key Laboratory of Molecular Biology, Shanghai Institute of Biochemistry and Cell Biology, Center for Excellence in Molecular Cell Science, Chinese Academy of Sciences; University of Chinese Academy of Sciences, Shanghai 200031, China

[2]Key Laboratory of Systems Health Science of Zhejiang Province, School of Life Science, Hangzhou Institute for Advanced Study, University of Chinese Academy of Sciences, Hangzhou 310024, China

[3]National Institute of Biological Sciences, Beijing 102206, China

[4]Shanghai Key Laboratory of Brain Function Restoration and Neural Regeneration, Huashan Hospital, Shanghai Medical College, Fudan University, Shanghai 200032, China

[*]Correspondence and requests for materials should be addressed to F.H. (email: fhou@sibcb.ac.cn)

Supplementary Figure 1-8

Supplementary Figure 1

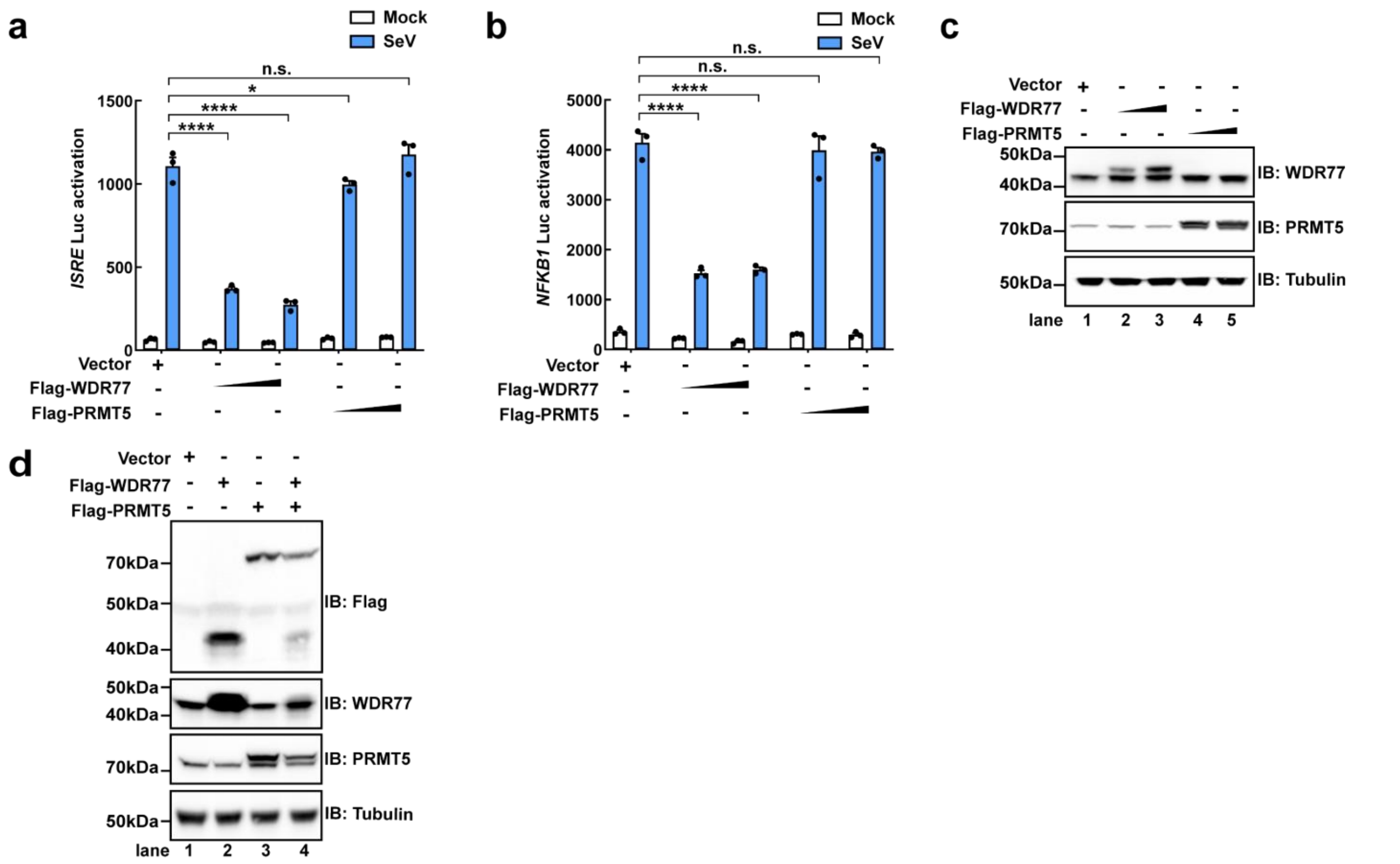

Supplementary Figure 1 │ WDR77 negatively regulates RIG-I-MAVS signaling pathway.

a-**c** HEK293T cells were transfected with luciferase reporters and increasing amounts of Flag-WDR77 or PRMT5-expressing plasmids for 24 h, and then stimulated with or without SeV for 12 h. Cells were harvested and *ISRE* (**a**), *NFKB1* (**b**) promotor activation was detected by luciferase assay. Protein expression levels were detected by immunoblotting (**c**) (For **a**, *ISRE*: ****$p < 0.0001$, ****$p < 0.0001$, *$p = 0.0324$, $^{ns}p = 0.2374$ in sequence. For **b**, *NFKB1*: **** $p < 0.0001$, ****$p < 0.0001$, $^{ns}p = 0.7305$, $^{ns}p = 0.6305$ in sequence.). **d** Immunoblot analysis of the expression level of Flag-WDR77, PRMT5 as indicated in Fig. 1**i**. Data are representative of three independent experiments with similar results (**c** and **d**), or three independent experiments (**a** and **b**) (mean ± SD of three biological replicates). *P* values were determined by two-way ANOVA (Dunnett's test) (**a**, **b**). n.s. indicates no statistical significance. Source data are provided as a Source Data file.

Supplementary Figure 2

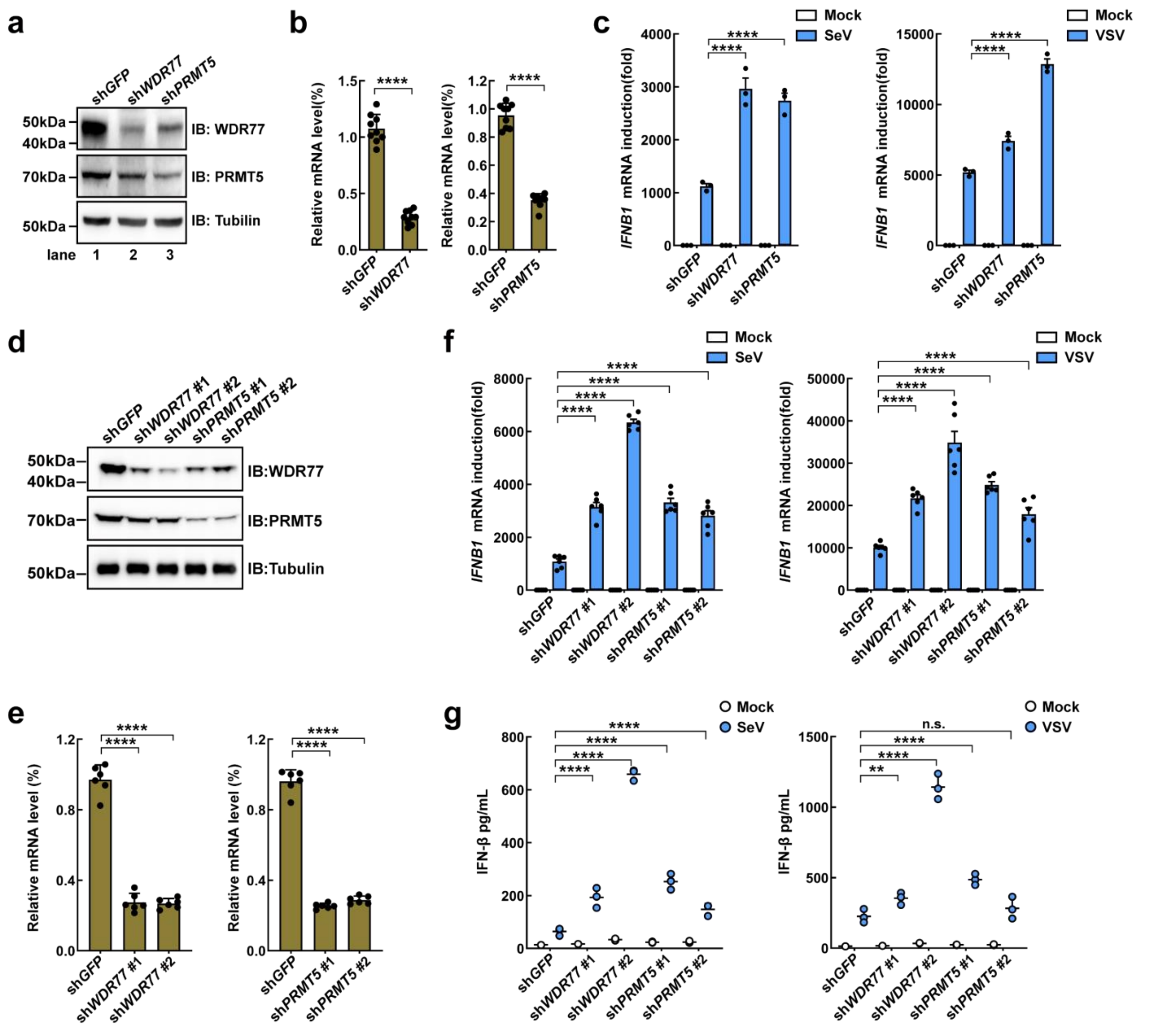

Supplementary Figure 2 │ Knockdown of WDR77 promotes IFN-β induction.

a-**c** shRNA targeting WDR77 or PRMT5 was transduced into HEK293T cells for 24 h. Cells were treated with puromycin (1 μg/ml) for 48 h and infected with SeV or VSV for 12 h. Whole cell lysates were subjected to immunoblotting (**a**). Knock down efficiency (**b**) and *IFNB1* induction (**c**) were measured by qPCR (For **b**, knockdown efficiencies: all ****p < 0.0001; For **c**, *IFNB1*: all ****p < 0.0001). **d-e** WDR77 and PRMT5 expression was detected by immunoblotting in stable knockdown HEK293T cell lines (**d**). Knock down efficiency were also measured by qPCR (**e**) (Knockdown efficiencies: all ****p < 0.0001). **f-g** sh*GFP*, sh*WDR77* or sh*PRMT5* HEK293T cells were stimulated

with SeV or VSV for 12 h before *IFNB1* induction was measured by qPCR (**f**). Culture medium was collected and IFN-β was detected by ELISA (**g**) (For **f**, *IFNB1*: all ****$p < 0.0001$; For **g**, IFN-β SeV: all ****$p < 0.0001$; IFN-β VSV: **$p = 0.0088$; ****$p < 0.0001$; ****$p < 0.0001$; $^{ns}p = 0.4337$ in sequence). Data are representative of two independent experiments with similar results (**a** and **d**), or three independent experiments (shown as mean ± SD in **b**, **c**, and **e**-**g**) (n = 9 in **b**, n = 3 in **c** and **g**, or n = 3 with 2 technical replicates in **e** and **f**). *P* values were determined by unpaired two-sided *t*-test (**b**), ordinary one-way ANOVA (Dunnett's test) (**e**) or two-way ANOVA (Šídák's test) (**c**, **f**, **g**). n.s. indicates no statistical significance. Source data are provided as a Source Data file.

Supplementary Figure 3

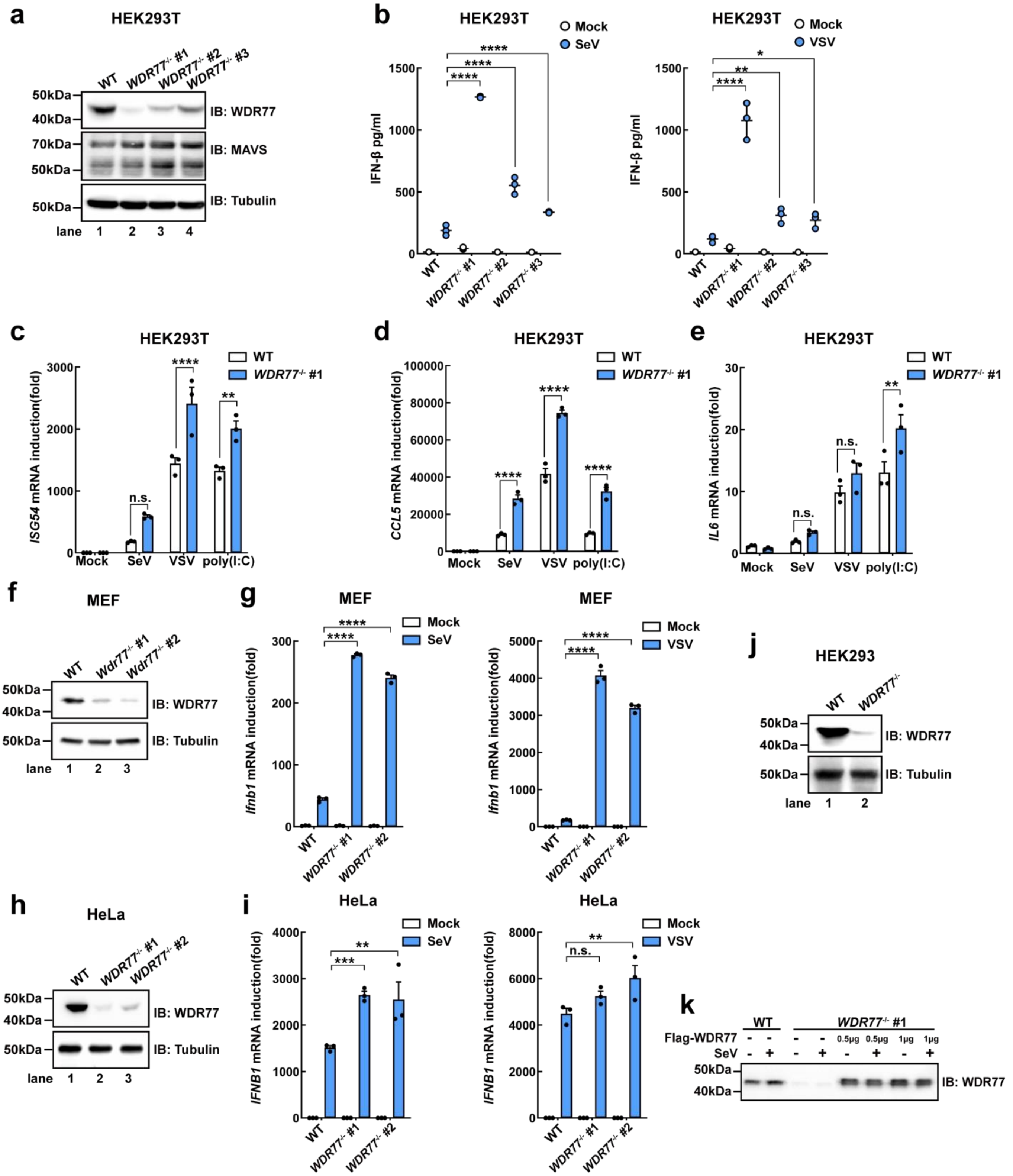

Supplementary Figure 3 │ Deficiency of WDR77 promotes IFN-β induction.

a WDR77 expression was detected by immunoblotting in three *WDR77* knockout cell lines. **b** WT or *WDR77*$^{-/-}$ HEK293T cells were stimulated with SeV or VSV for 12 h. Culture medium was collected

and IFN-β was detected by ELISA (For **b**, IFN-β: all $^{****}p < 0.0001$, $^{**}p = 0.050$, $^{*}p = 0.0252$ in sequence). **c**-**e** WT or *WDR77*$^{-/-}$ #1 HEK293T cells were stimulated with SeV, VSV or poly(I:C) for 12 h. *ISG54* (**c**), *CCL5* (**d**) and *IL6* (**e**) induction were measured by qPCR (For **c**, *ISG54*: $^{ns}p = 0.0747$, $^{****}p < 0.0001$, $^{**}p = 0.0019$ in sequence; For **d**, *CCL5*: all $^{****}p < 0.0001$; For **e**, *IL6*: $^{ns}p = 0.8665$, $^{ns}p = 0.3027$, $^{**}p = 0.0027$ in sequence). **f** Immunoblot analysis of WDR77 in WT and *Wdr77*$^{-/-}$ MEF cells. **g** WT or *Wdr77*$^{-/-}$ MEF cells were stimulated with SeV and VSV for 12 h, *Ifnb1* induction was measured by qPCR (*Ifnb1*: all $^{****}p < 0.0001$). **h** Immunoblot analysis of WDR77 in wild-type and *WDR77*$^{-/-}$ HeLa cells. **i** WT or *WDR77*$^{-/-}$ HeLa cells were stimulated with SeV or VSV for 12 h. *IFNB1* induction was measured by qPCR (*IFNB1*: $^{***}p = 0.0006$; $^{**}p = 0.0012$; $^{ns}p = 0.1060$; $^{**}p = 0.0021$ in sequence). **j** Immunoblot analysis of WDR77 in WT and *WDR77*$^{-/-}$ HEK293 cells. **k** WT or *WDR77*$^{-/-}$ #1 HEK293T were transfected with increasing amounts of WDR77-expressing plasmids for 24 h, and then stimulated with or without SeV for 12 h. Protein expression levels were detected by immunoblotting. Data are representative of two independent experiments with similar results (**a**, **f**, **h**, **j** and **k**), or three independent experiments (**b**-**e**, **g** and **i**) (mean ± SD of three biological replicates). *P* values were determined by two-way ANOVA (Šídák's test) (**b**-**e**, **g**, **i**). n.s. indicates no statistical significance. Source data are provided as a Source Data file.

Supplementary Figure 4

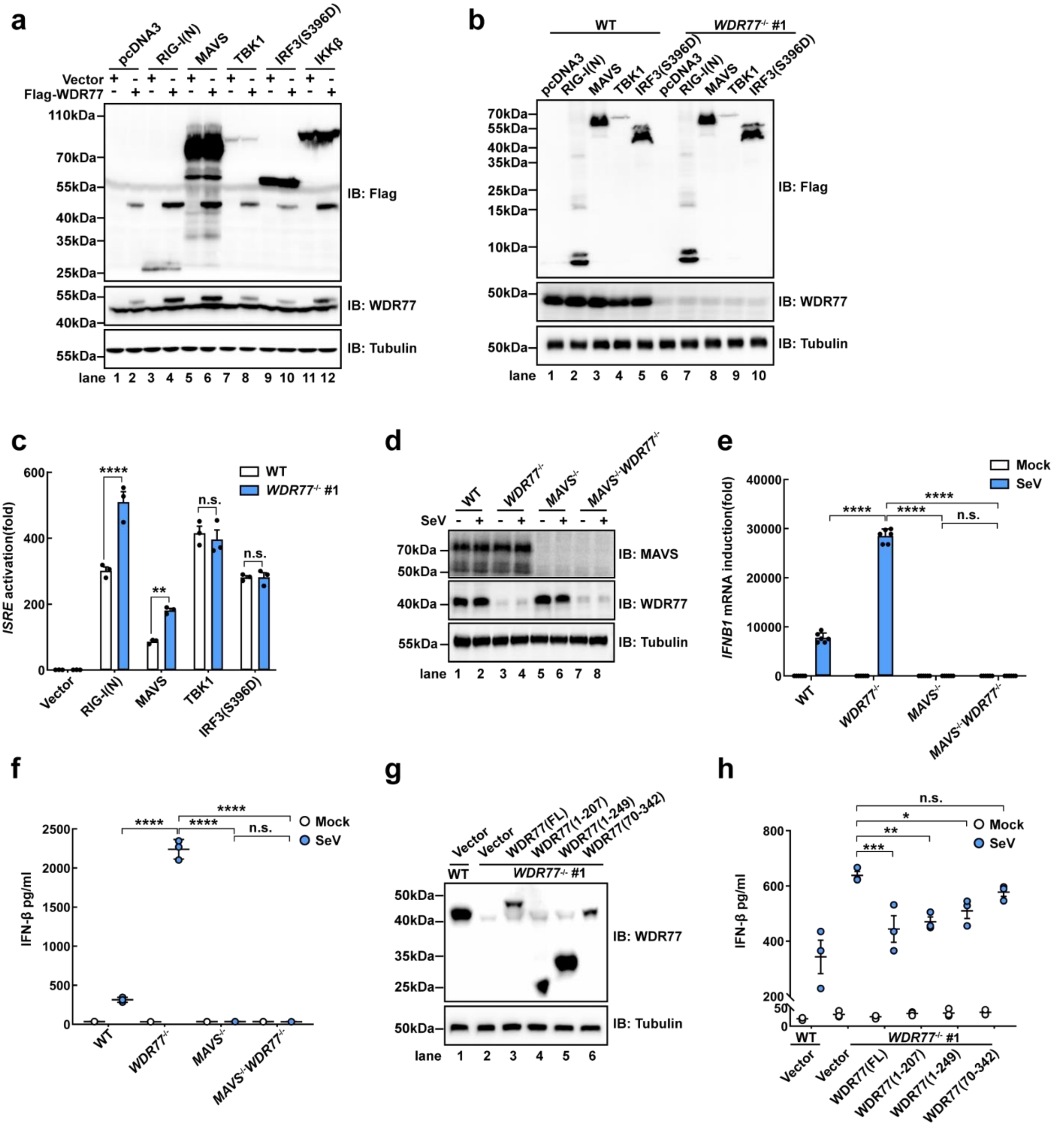

Supplementary Figure 4 │ WDR77 acts on or downstream of MAVS.

a Immunoblot analysis of Flag-RIG-I(N), MAVS, TBK1, IRF3(S396D) and IKKβ as indicated in Fig. 3**a**. **b**-**c** WT or *WDR77*$^{-/-}$ #1 HEK293T cells were transfected with luciferase reporters and various expression plasmids as indicated for 24 h. *ISRE* promotor activation was detected by luciferase assay (**c**), and the protein expression levels were detected by immunoblotting (**b**) (*ISRE*: ****p <

0.0001; **p = 0.0028; ^{ns}p = 0.9308; ^{ns}p > 0.9999 in sequence). **d-f** Various cell lines as indicated were stimulated with or without SeV for 12 h and cells were harvested for immunoblotting (**d**). *IFNB1* induction was measured by qPCR (**e**). Culture medium was collected and IFN-β was detected by ELISA (**f**) (For **e** and **f**, *IFNB1/* IFN-β: all ****p < 0.0001; ^{ns}p > 0.9999). **g-h** WT or *WDR77*$^{-/-}$ #1 HEK293T were transfected with various WDR77 truncations for 24 h, and then stimulated with or without SeV for 12 h. Cells were harvested for immunoblotting (**g**). Culture medium was collected and IFN-β was detected by ELISA (**h**) (IFN-β: ***p = 0.0002; **p = 0.0013; *p = 0.0215; ^{ns}p = 0.8036 in sequence). Data are representative of three independent experiments with similar results (**a**, **b**, **d** and **g**), or three independent experiments (**c**, **e**, **f** and **h**) (mean ± SD of three biological replicates, three biological replicates with two technical replicates in **e**). *P* values were determined by two-way ANOVA (Šídák's test) (**c**, **e**, **f**, **h**). n.s. indicates no statistical significance. Source data are provided as a Source Data file.

Supplementary Figure 5

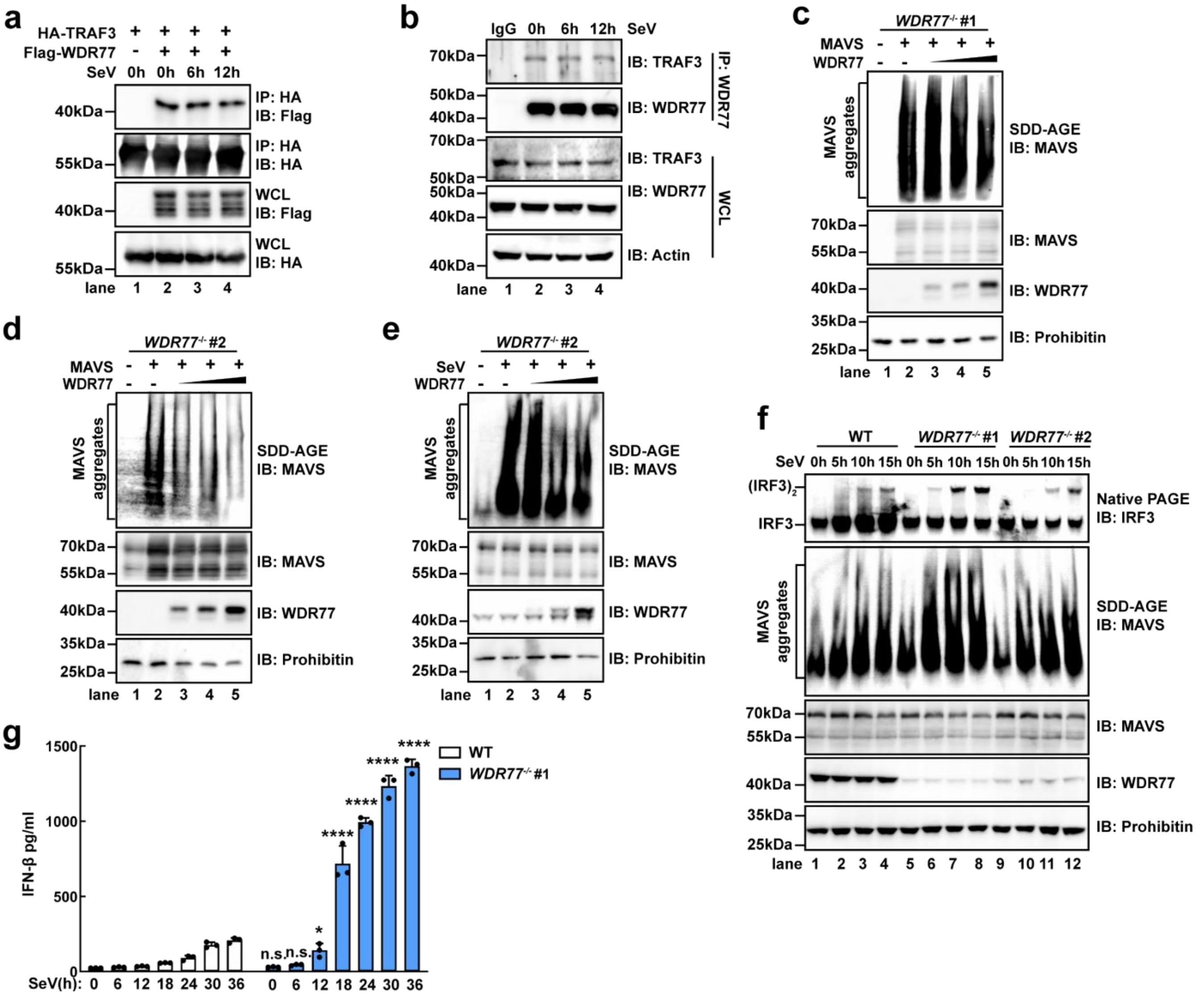

Supplementary Figure 5 │ WDR77 prevents MAVS aggregation.

a HEK293T cells were transfected with Flag-WDR77 and HA-TRAF3 for 24 h before stimulation with SeV. Cells were collected and subjected to immunoprecipitation assay and immunoblotting. **b** HEK293T cells were stimulated with SeV and harvested for immunoprecipitation assay and immunoblotting. **c**, **d** *WDR77*$^{-/-}$ #1 (**c**) or *WDR77*$^{-/-}$ #2 (**d**) HEK293T cells were transfected with increasing amounts of Flag-WDR77 plasmids for 24 h, followed by transfection with Flag-MAVS plasmids for 12 h. Cells were then harvested for subcellular fractionation. P5 fractions were subjected to SDD-AGE to examine MAVS aggregation. **e** *WDR77*$^{-/-}$ #2 HEK293T cells were transfected with increasing amounts of Flag-WDR77 plasmids for 24 h, and then stimulated with or without SeV for 8 h. Cells were harvested for subcellular fractionation. P5 fractions were subjected

to SDD-AGE to examine MAVS aggregation. **f** WT or *WDR77*$^{-/-}$ HEK293T cells were stimulated with SeV. At specific time points post infection as indicated, cells were collected for following subcellular fractionation. S5 fractions were subjected to native PAGE to examine IRF3 dimer, and P5 fractions were subjected to SDD-AGE to examine MAVS aggregation. **g** WT or *WDR77*$^{-/-}$ #1 HEK293T cells were stimulated with SeV. At specific time points post infection as indicated, culture medium was collected and IFN-β was detected by ELISA (IFN-β: $^{ns}p > 0.9999$; $^{ns}p = 0.9995$; $^{*}p = 0.0301$, all $^{****}p < 0.0001$ in sequence). Data are representative of three independent experiments with similar results (**a**- **f**), or three independent experiments (**g**) (mean ± SD of three biological replicates). *P* values were determined by two-way ANOVA (Šídák's test) (**g**). n.s. indicates no statistical significance. Source data are provided as a Source Data file.

Supplementary Figure 6

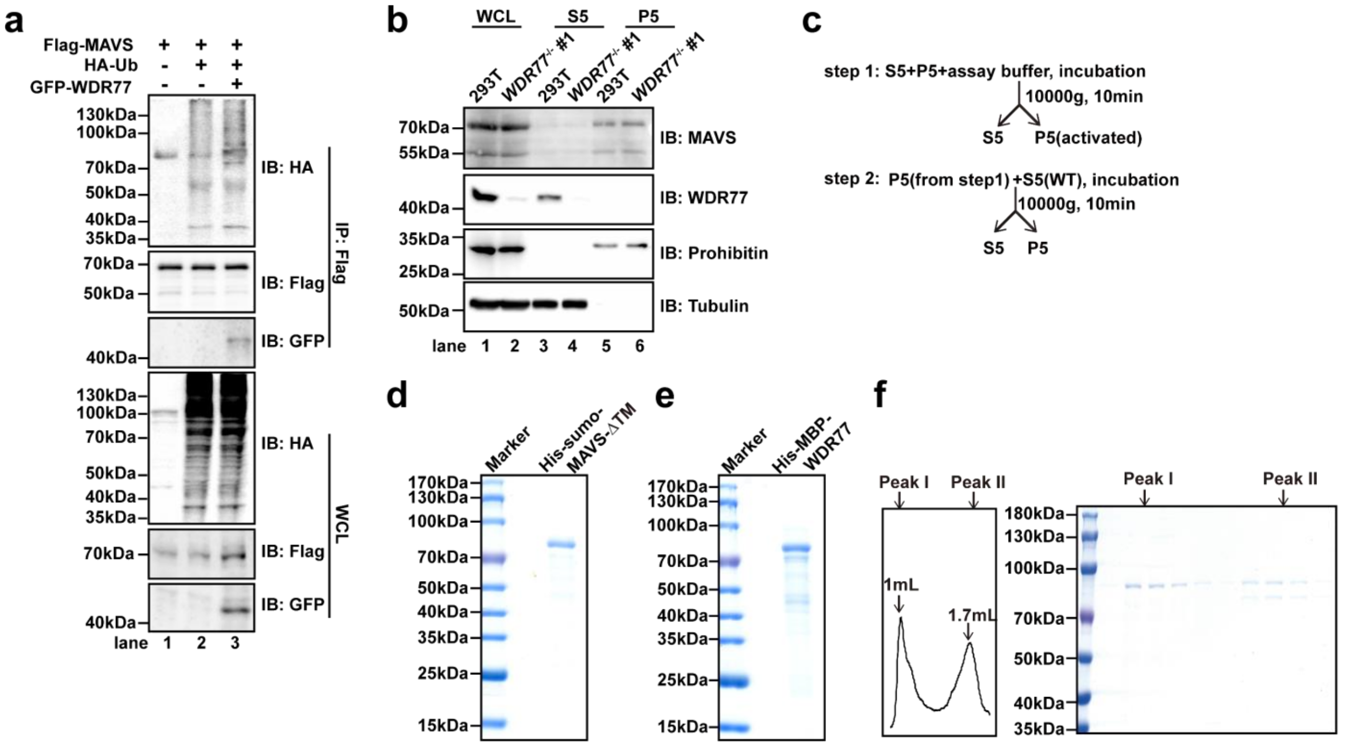

Supplementary Figure 6 │ Supplementary information for Figure 5.

a HEK293T cells were co-transfected with Flag-MAVS, HA-Ub and GFP-WDR77 as indicated for 24 h. Cells were then harvested and subjected to immunoprecipitation assay and immunoblotting. **b** Immunoblot analysis of S5 and P5 fractions from WT and *WDR77*$^{-/-}$ #1 HEK293T cells. **c** Procedures of the two-step assay. In step 1, S5 fraction was incubated with the P5 fraction, and P5 was isolated. In step 2, P5 from step 1 was incubated with fresh S5 followed by immunoblotting as indicated. **d** Coomassie blue staining of purified His-Sumo-MAVS-ΔTM following SDS-PAGE. **e** Coomassie blue staining of purified His-MBP-WDR77 following SDS-PAGE. **f** His-Sumo-MAVS-ΔTM was separated into two fractions (Peak-I and Peak-II) over a Superdex-200 column (left). Recombinant MAVS was visualized by Coomassie blue staining following SDS-PAGE (right). Data are representative of three independent experiments with similar results (**a**-**b**, **d**-**f**). Source data are provided as a Source Data file.

Supplementary Figure 7

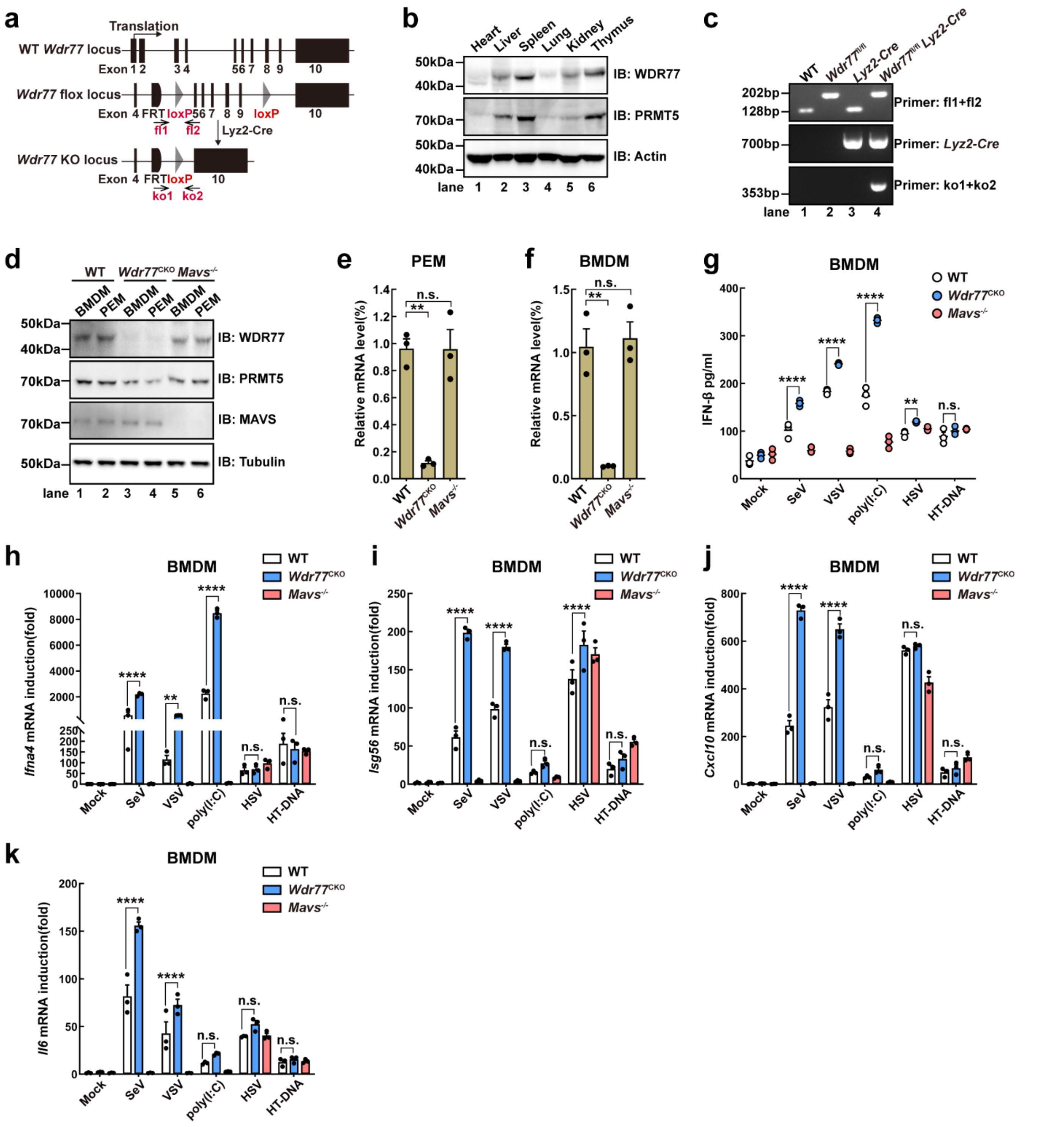

Supplementary Figure 7 │ WDR77 deficiency in primary macrophages enhances RLR-mediated IFN signaling.

a Construction strategy of *Wdr77*$^{fl//fl}$ mice. **b** Immunoblot analysis of WDR77 expression in mouse heart, liver, spleen, lung or thymus. **c** Genotyping results of *Wdr77*$^{fl//fl}$ *Lyz2-Cre* mice. **d** Immunoblot

analysis of WDR77 expression in BMDMs and PEMs from WT, *Wdr77*^{CKO} and *Mavs*$^{-/-}$ mice. **e**, **f** qPCR analysis of *Wdr77* in PEMs (**e**) and BMDMs (**f**) from WT, *Wdr77*^{CKO} and *Mavs*$^{-/-}$ mice (For **e**, knockdown efficiency: $^{**}p$ =0.0013, ^{ns}p = 0.9993; For **f**, knockdown efficiency: $^{**}p$ = 0.0016, ^{ns}p = 0.8777). **g-k** BMDMs from WT, *Wdr77*^{CKO} and *Mavs*$^{-/-}$ mice were stimulated for 6 h with or without VSV, SeV, poly(I:C), HSV, or HT-DNA. Culture mediums were then collected, and IFN-β was measured by ELISA (**g**). *Ifna4* (**h**), *Cxcl10* (**i**), *Isg56* (**j**), *Il-6* (**k**) induction were measured by qPCR (For **g**, IFN-β: all $^{****}p$ < 0.0001, $^{**}p$ = 0.0059, ^{ns}p = 0.2387 in sequence; For **h**, *Ifna4*: $^{****}p$ < 0.0001, $^{**}p$ = 0.0049, $^{****}p$ < 0.0001, ^{ns}p > 0.9999, ^{ns}p = 0.9967 in sequence; For **i**, *Isg56*: all $^{****}p$ < 0.0001, ^{ns}p = 0.3319, ^{ns}p = 0.2791 in sequence; For **j**, *Cxcl10*: all $^{****}p$ < 0.0001, ^{ns}p = 0.2386, ^{ns}p = 0.5527, ^{ns}p = 0.5939 in sequence; For **k**, *Il-6*: all $^{****}p$ < 0.0001, ^{ns}p = 0.2432,, ^{ns}p = 0.0968, ^{ns}p = 0.8685 in sequence). Data are representative of three independent experiments with similar result (**b- d**), or three independent experiments (**e** to **k**) (mean ± SEM of three biological replicates). *P* values were determined by ordinary one-way ANOVA (Dunnett's test) (**e**, **f**), two-way ANOVA (Dunnett's test) (**g**, **i**-**k**) or two-way ANOVA (Šídák's test) (**h**). n.s. indicates no statistical significance. Source data are provided as a Source Data file.

Supplementary Figure 8

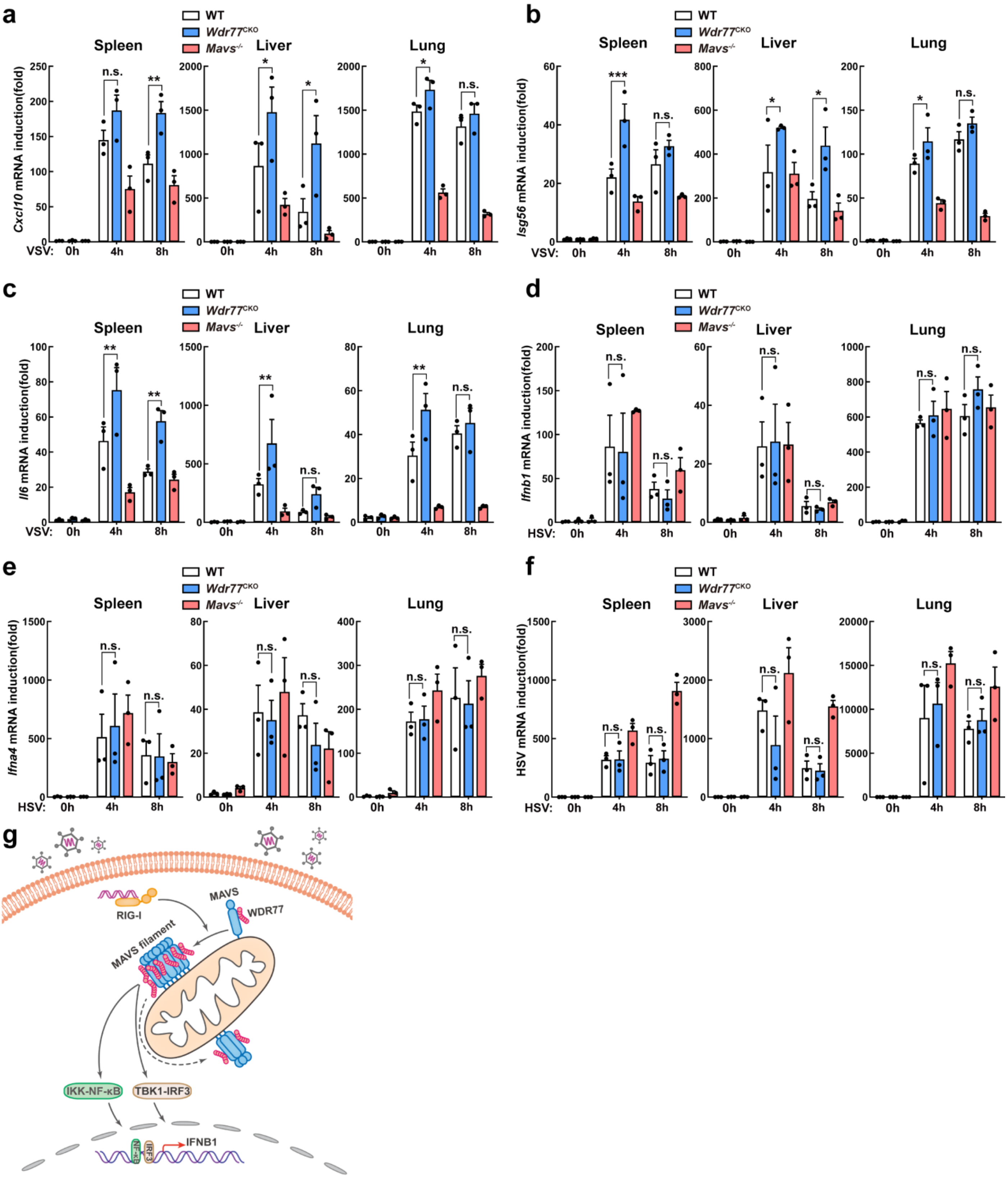

Supplementary Figure 8 │ WDR77 deficiency potentiates host defense against RNA virus in mice.

a-c WT, *Wdr77*^{CKO} and *Mavs*$^{-/-}$ mice (n=3 each) were infected intravenously with VSV at 2×10^{7} PFU

per mouse. The spleens, livers and lungs were collected 4 h or 8 h after infection. *Cxcl10* (**a**), *Isg56* (**b**) and *Il6* (**c**) inductions were measured respectively by qPCR (For **a**, *Cxcl10*: ^{ns}p = 0.0701, $^{**}p$ = 0.0023, $^{*}p$ = 0.0434, $^{*}p$ = 0.0107, $^{*}p$ = 0.0346, ^{ns}p = 0.2504 in sequence; For **b**, *Isg56*: $^{***}p$ = 0.0002, ^{ns}p =0.2780, $^{*}p$ = 0.0445, $^{*}p$ = 0.0157, $^{*}p$ =0.0433, ^{ns}p = 0.1715 in sequence; For **c**, *Il6*: $^{**}p$ = 0.0034, $^{**}p$ = 0.0035, $^{**}p$ = 0.0063, ^{ns}p =0.2648, $^{**}p$ = 0.0037, ^{ns}p =0.6250 in sequence). **d**-**f** WT, *Wdr77*^{CKO} and *Mavs*$^{-/-}$ mice (n=3 each) were infected intravenously with HSV at 1.5×10^8 PFU per mouse. The spleens, livers, and lungs were collected 4 h or 8 h post infection. *Ifnb1* (**d**), *Ifna4* (**e**) inductions and HSV RNA levels (**f**) were measured respectively by qPCR (For **d**, *Ifnb1*: ^{ns}p = 0.9760, 0.9218, 0.9707, 0.9847, 0.8515, 0.1808 in sequence; For **e**, *Ifna4*: ^{ns}p = 0.8904, 0.9988, 0.9883, 0.6251, 0.9921,0.9445 in sequence; For **f**, HSV: ^{ns}p = 0.9990, 0.8555, 0.2076, 0.9904, 0.7482, 0.8981 in sequence). **g** A schematic model illustrating the functional involvement of WDR77 in RIG-I-MAVS-mediated antiviral signaling pathway. Data are representative of three independent experiments (**a** to **f**) (mean ± SEM of three biological replicates). *P* values were determined by two-way ANOVA (Dunnett's test) (**a**, **c**, **d** (liver), **e** (lung), **f** (spleen, lung)), two-way ANOVA (Tukey's test) (**b**, **d** (spleen, lung), **e** (spleen), **f** (liver)), or two-way ANOVA (Šídák's test) (**e** (liver)). n.s. indicates no statistical significance. Source data are provided as a Source Data file.

Supplementary Figure 1, Western blot images of the whole membranes

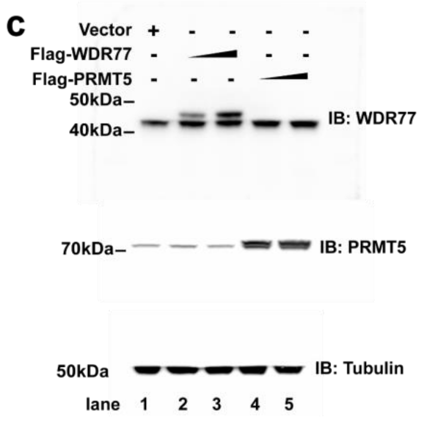

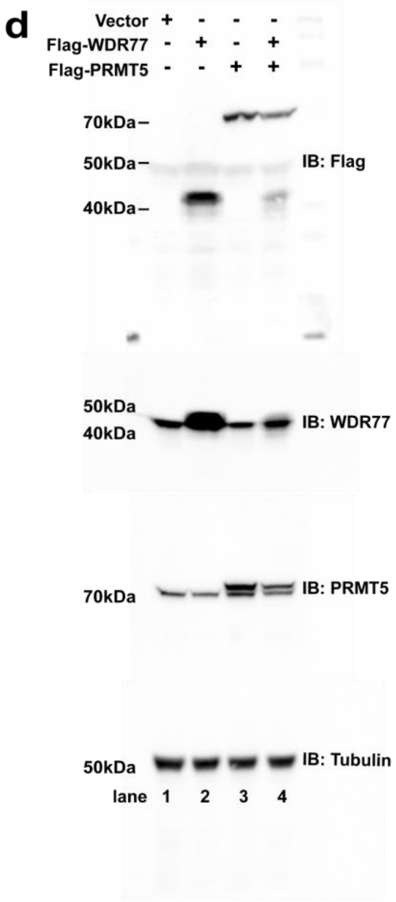

Supplementary Figure 2, Western blot images of the whole membranes

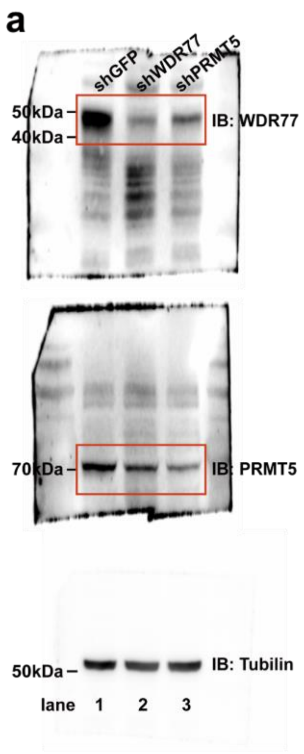

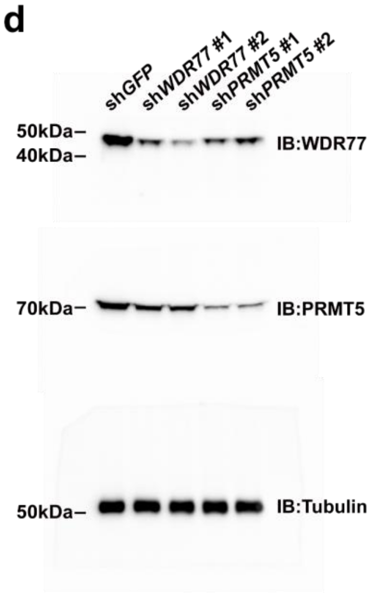

Supplementary Figure 3, Western blot images of the whole membranes

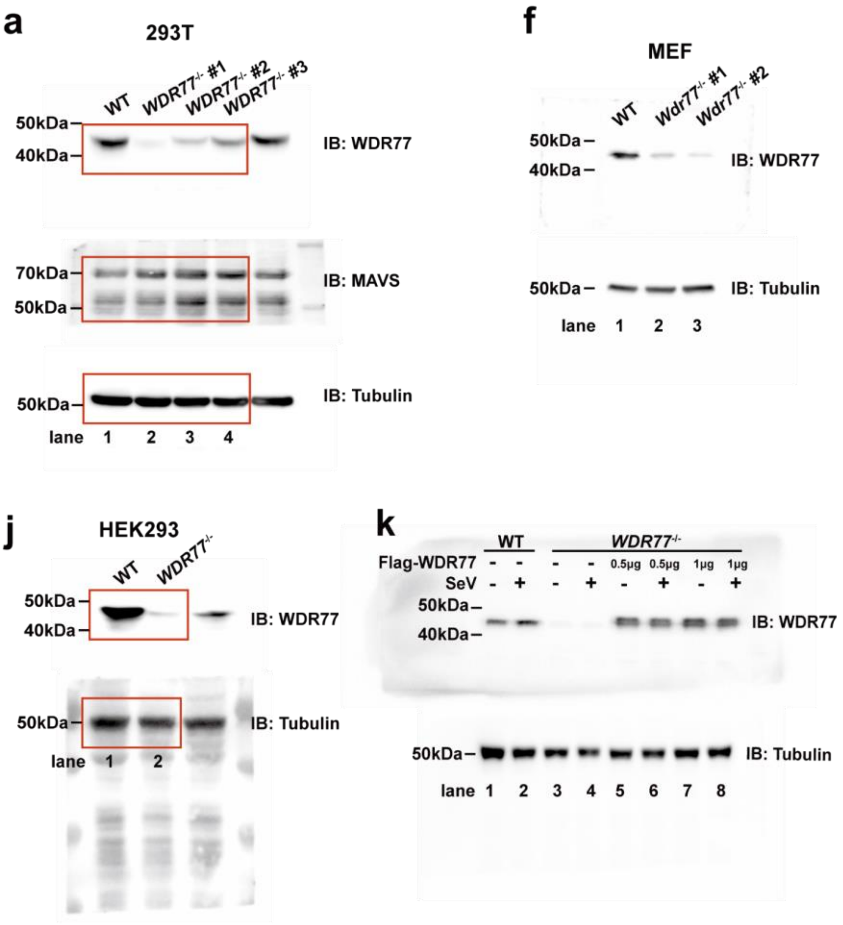

Supplementary Figure 4, Western blot images of the whole membranes

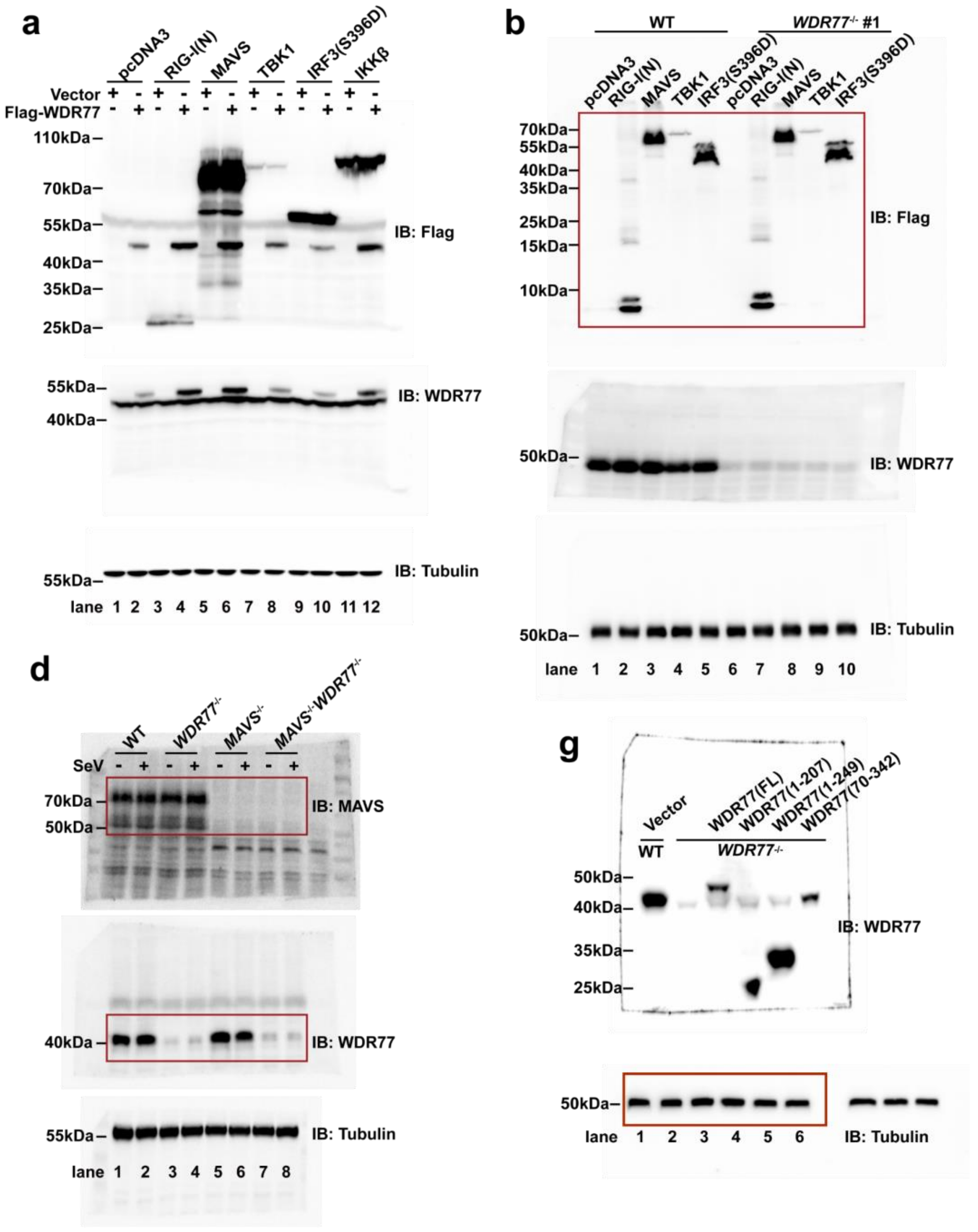

Supplementary Figure 5, Western blot images of the whole membranes

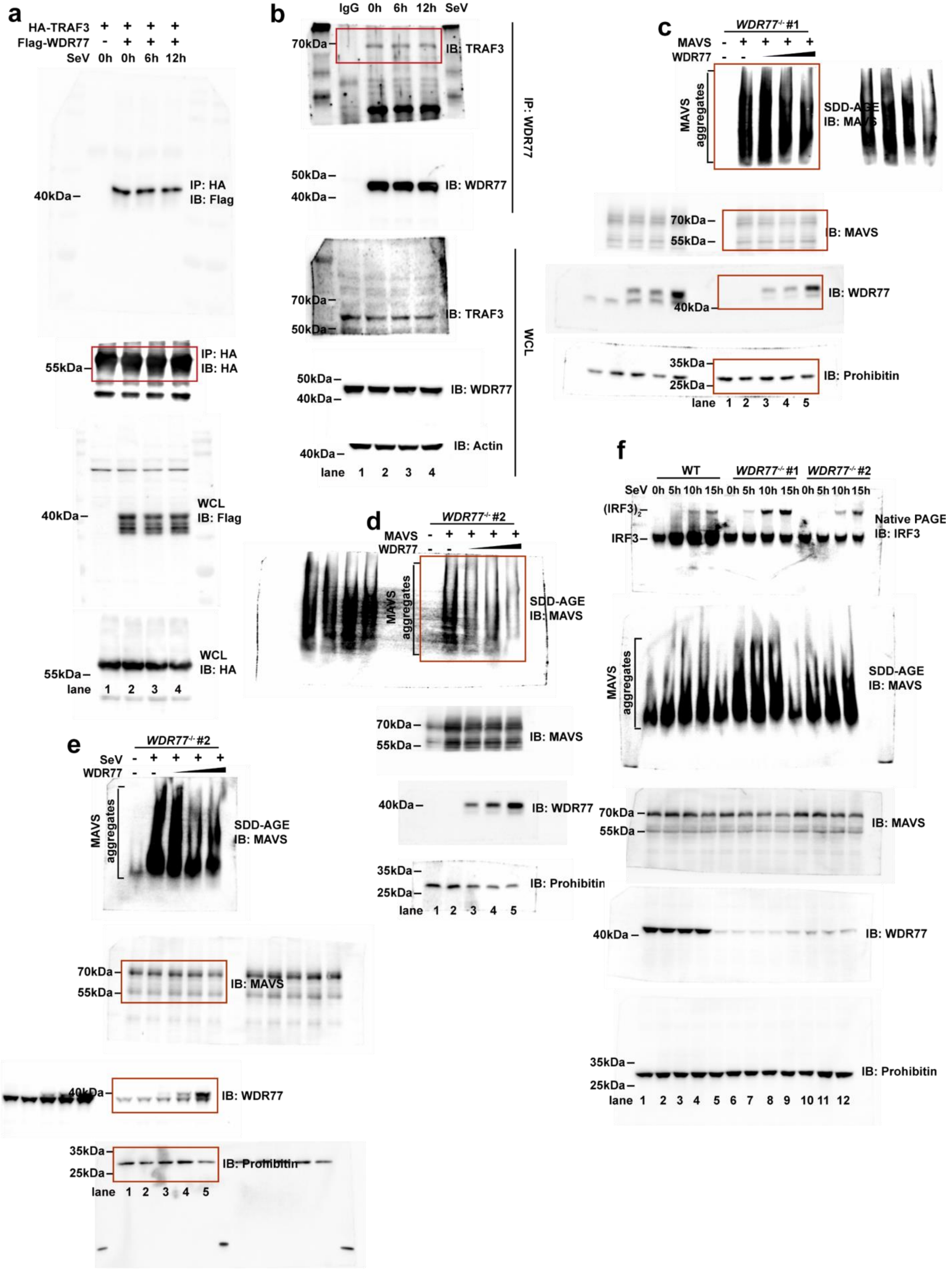

Supplementary Figure 6, Western blot images of the whole membranes

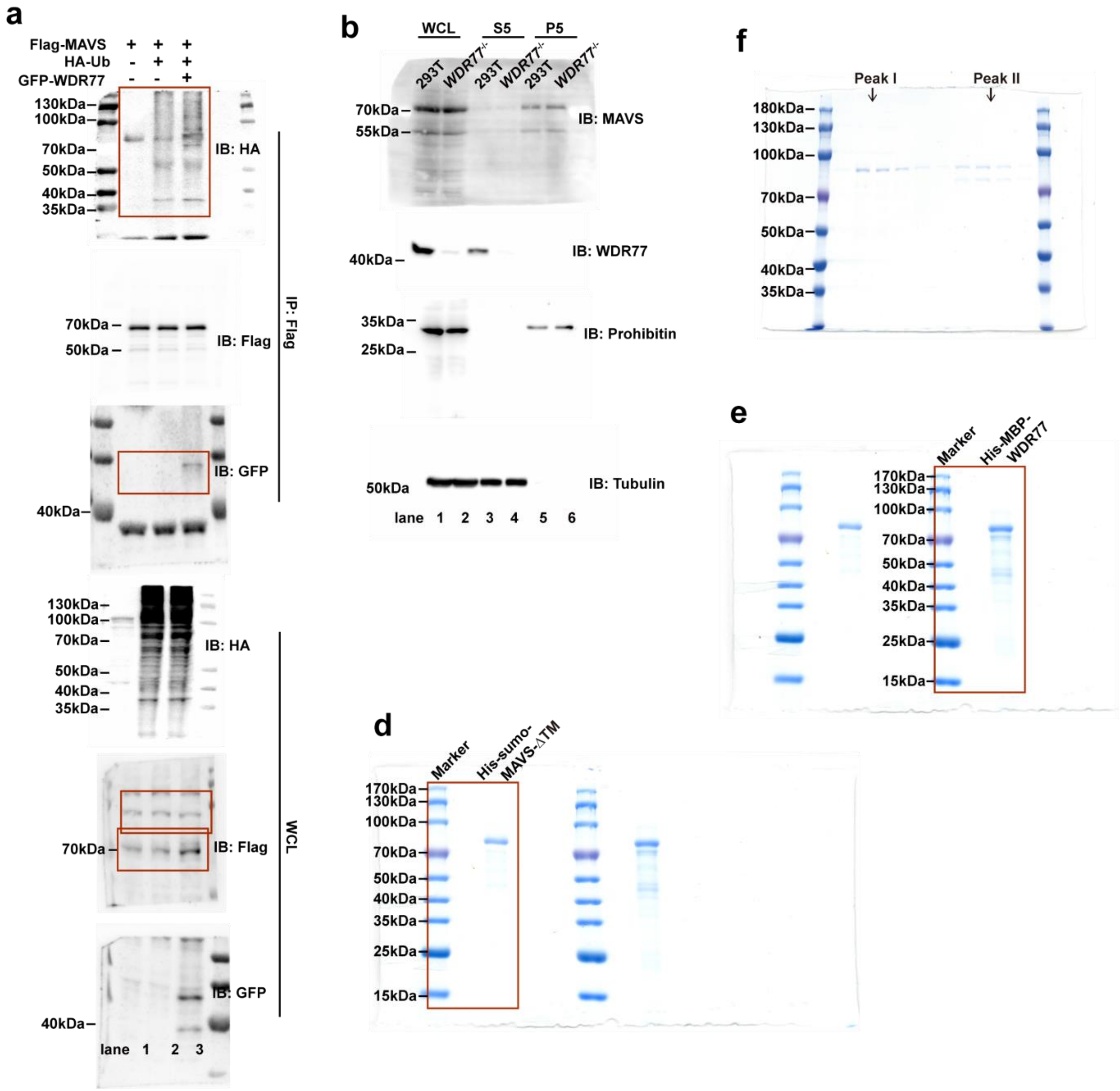

Supplementary Figure 7, Western blot images of the whole membranes

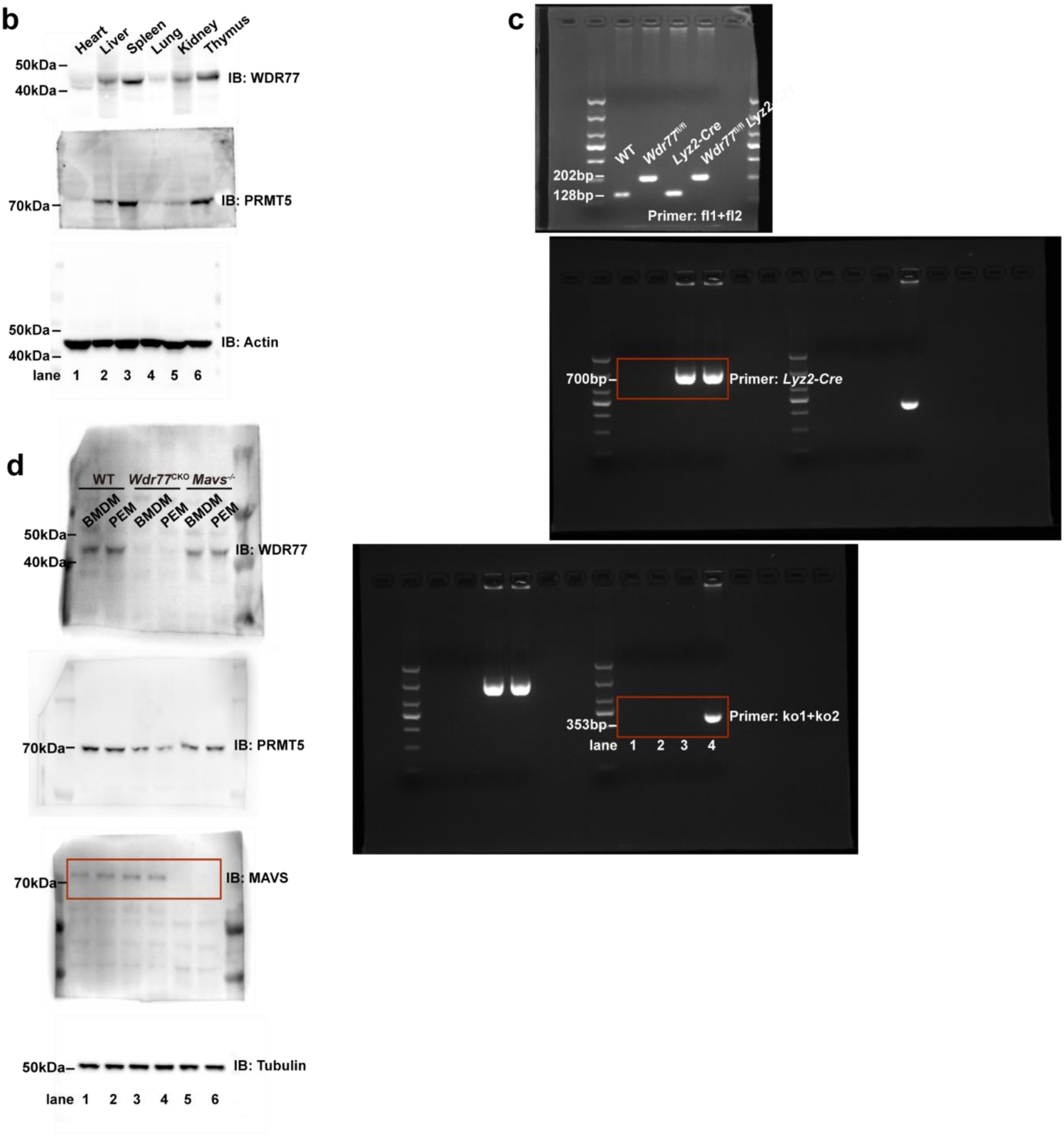

Supplement: Supplementary file 1 — Supplementary Information [file 41467_2023_40567_MOESM1_ESM.pdf]
