# Supplementary material for: WDR77 inhibits prion-like aggregation of MAVS to limit antiviral innate immune response
Source: Nat Commun. 2023 Aug 10;14:4824. doi: 10.1038/s41467-023-40567-5 (PMC10415273; doi:10.1038/s41467-023-40567-5)
Supplement: Supplementary file 3 — Description of Additional Supplementary Files [file 41467_2023_40567_MOESM3_ESM.pdf]

Description of Additional Supplementary Files

File Name: Supplementary Data 1
Description: Sequence of oligoes for molecular cloning, Cas9-mediated gene editing or mRNA knockdown.
