# Supplementary material for: WDR77 inhibits prion-like aggregation of MAVS to limit antiviral innate immune response
Source: Nat Commun. 2023 Aug 10;14:4824. doi: 10.1038/s41467-023-40567-5 (PMC10415273; doi:10.1038/s41467-023-40567-5)
Supplement: Supplementary file 5 — Reporting Summary [file 41467_2023_40567_MOESM5_ESM.pdf]

nature portfolio

Corresponding author(s): Fajian Hou

Last updated by author(s): Jul 18, 2023

Reporting Summary

Nature Portfolio wishes to improve the reproducibility of the work that we publish. This form provides structure for consistency and transparency in reporting. For further information on Nature Portfolio policies, see our Editorial Policies and the Editorial Policy Checklist.

Statistics

For all statistical analyses, confirm that the following items are present in the figure legend, table legend, main text, or Methods section.

n/a	Confirmed	
☐	☒	The exact sample size (*n*) for each experimental group/condition, given as a discrete number and unit of measurement
☐	☒	A statement on whether measurements were taken from distinct samples or whether the same sample was measured repeatedly
☐	☒	The statistical test(s) used AND whether they are one- or two-sided<br>*Only common tests should be described solely by name; describe more complex techniques in the Methods section.*
☒	☐	A description of all covariates tested
☐	☒	A description of any assumptions or corrections, such as tests of normality and adjustment for multiple comparisons
☐	☒	A full description of the statistical parameters including central tendency (e.g. means) or other basic estimates (e.g. regression coefficient) AND variation (e.g. standard deviation) or associated estimates of uncertainty (e.g. confidence intervals)
☐	☒	For null hypothesis testing, the test statistic (e.g. *F*, *t*, *r*) with confidence intervals, effect sizes, degrees of freedom and *P* value noted<br>*Give P values as exact values whenever suitable.*
☒	☐	For Bayesian analysis, information on the choice of priors and Markov chain Monte Carlo settings
☒	☐	For hierarchical and complex designs, identification of the appropriate level for tests and full reporting of outcomes
☒	☐	Estimates of effect sizes (e.g. Cohen's *d*, Pearson's *r*), indicating how they were calculated

Our web collection on statistics for biologists contains articles on many of the points above.

Software and code

Policy information about availability of computer code

Data collection	1. qRT-PCR data were collected on LightCycler-® 480 Instrument II (Roche) and analysed using the LightCycler480 software version 1.5 (Roche).<br>2. Western blot were visualized by the MiniChemTM 610 Plus (Sagecreation) using the SageCaptureTM software version 2.19.12 (Sagecreation).<br>3. Renilla Luciferase activity was analyzed by GloMax® 20/20 Luminometer (Promega) using GloMax® 20/20 Luminometer software version 1.10 (Promega).<br>4. Immunofluorescence images were acquired on Leica TCS SP8 STED X (Leica) using Leica Application Suite X (LAS X) software version 1.4.4 (Leica).<br>5. Transmission electron microscopy images were acquired on Tecnai™ G2 Spirit (FEI) using Ganta CCD camera.
Data analysis	1. Quantification of Western blot was performed on ImageJ version 1.52a.<br>2. Western blot images and micrograph images were assembled in Adobe Illustrator CC 2019 version 23.0.02.<br>3. qRT-PCR data was analyzed in Microsoft Excel 2021 version 16.0.16529.20164.<br>4. Statistical analysis was done in Prims version 9 (Graphpad) or Microsoft Excel 2021 version 16.0.16529.20164.

For manuscripts utilizing custom algorithms or software that are central to the research but not yet described in published literature, software must be made available to editors and reviewers. We strongly encourage code deposition in a community repository (e.g. GitHub). See the Nature Portfolio guidelines for submitting code & software for further information.

Data

Policy information about availability of data

All manuscripts must include a data availability statement. This statement should provide the following information, where applicable:

- Accession codes, unique identifiers, or web links for publicly available datasets
- A description of any restrictions on data availability
- For clinical datasets or third party data, please ensure that the statement adheres to our policy

The authors declare that all data supporting the finding of this study are available within the article and its supplementary information files. Source data for figures 1-7 and supplementary figures 1-8 are provided with the paper.

Research involving human participants, their data, or biological material

Policy information about studies with human participants or human data. See also policy information about sex, gender (identity/presentation), and sexual orientation and race, ethnicity and racism.

Reporting on sex and gender	N/A
Reporting on race, ethnicity, or other socially relevant groupings	N/A
Population characteristics	N/A
Recruitment	N/A
Ethics oversight	N/A

Note that full information on the approval of the study protocol must also be provided in the manuscript.

Field-specific reporting

Please select the one below that is the best fit for your research. If you are not sure, read the appropriate sections before making your selection.

☒ Life sciences ☐ Behavioural & social sciences ☐ Ecological, evolutionary & environmental sciences

For a reference copy of the document with all sections, see nature.com/documents/nr-reporting-summary-flat.pdf

Life sciences study design

All studies must disclose on these points even when the disclosure is negative.

Sample size	In general, no calculations were done to determine sample size. The sample size was determined based on standards for experimental cell biology and animal studies, attempting to have a minimum of N = 3 biological replicates with sufficient reproducibility.<br>Although no sample size calculation was conducted, we believe that the chosen sample size is sufficient for the following reasons: Firstly, our research question is specific and well-defined, and we have identified a reasonable sample range through literature review to detect the effects of interest. Secondly, our experimental design and statistical analysis methods have been justified, validated, and applied in numerous similar studies.<br>The exact n numbers used in each experiment are indicated in the figure legends.
Data exclusions	No data were excluded from the analysis.
Replication	Experimental findings were reliably reproduced within our lab. Results shown are technical replicates from representative biological replicates. The information of replication is clearly found in the Figure legends.
Randomization	No statistical methods were used for randomization. For in vitro experiments, mouse BMDMs and PEMs were isolated from random wild-type or KO mice, then the isolated primary cells were allocated randomly into two and treated with the indicated stimulations or left untreated (control). For in vivo experiments, wild-type or KO mice were randomly allocated into experimental groups.
Blinding	Data was analyzed by software with objective outcomes and quantification was performed in an uniform manner for all samples tested. Therefore, blinding was not relevant for this study.

Reporting for specific materials, systems and methods

We require information from authors about some types of materials, experimental systems and methods used in many studies. Here, indicate whether each material, system or method listed is relevant to your study. If you are not sure if a list item applies to your research, read the appropriate section before selecting a response.

Materials & experimental systems

n/a	Involved in the study
☐	☒ Antibodies
☐	☒ Eukaryotic cell lines
☒	☐ Palaeontology and archaeology
☐	☒ Animals and other organisms
☒	☐ Clinical data
☒	☐ Dual use research of concern
☒	☐ Plants

Methods

n/a	Involved in the study
☒	☐ ChIP-seq
☒	☐ Flow cytometry
☒	☐ MRI-based neuroimaging

Antibodies

Antibodies used

Immunofluorescence
Primary Antibodies (Target; Clone; Brand; Fluorchorme and corresponding catalog number; dilution)
mouse anti-human Flag; M2; Sigma-Aldrich; unconjugated Cat#F3165; 1:200
rabbit anti-human HA; C29F4; Cell Signaling; unconjugated Cat#3724S; 1:200

Secondary Antibodies (Target; Clone; Brand; Fluorchorme and corresponding catalog numberr; dilution)
goat anti-rabbit IgG; polyconal; Invitrogen; Alexa Fluor™ 488 Cat#A11034; 1:1,000
goat anti-mouse IgG; polyconal; Jackson ImmunoResearch; Cyanine Cy™3; Cat#115-165-146; 1:1,000

Western blot
Primary Antibodies (Target; Clone; Brand; Fluorchorme and corresponding catalog numberr; dilution)
rabbit anti- human MAVS; polyconal; This laboratory; unconjugated; 1:10,000
mouse anti-human Flag; M2; Sigma-Aldrich; unconjugated Cat#F3165; 1:5,000
rabbit anti-human Flag; polyclonal; Millipore; unconjugated Cat# F7425; 1:5,000
rabbit anti-human HA; C29F4; Cell Signaling; unconjugated Cat#3724S; 1:2,000
mouse anti-human Tubulin; B-5-1-2; Sigma-Aldrich; unconjugated Cat#T5168; 1:7,500
rabbit anti-human TBK1; D1B4; Cell Signaling; unconjugated Cat#3504S; 1:1,000
rabbit anti-human Phospho-TBK1/NAK (Ser172); D52C2; Cell Signaling; unconjugated Cat#5483S; 1:1,000
rabbit anti-human Rig-I; D14G6; Cell Signaling; unconjugated Cat#3743S; 1:1,000
rabbit anti-human Phospho-IRF-3 (Ser386); E7J8G; Cell Signaling; unconjugated Cat#37829; 1:1,000
rabbit anti-mouse MAVS; polyconal; Cell Signaling; unconjugated Cat#4983S; 1:1,000
rabbit anti-human IRF3; polyconal; Proteintech; unconjugated Cat#11312-1-AP; 1:2,000
rabbit anti-human TRAF3; polyconal; Santa Cruz; unconjugated Cat#sc-1828; 1:1,000
rabbit anti-human Prohibitin; EP2803Y; Abcam; unconjugated Cat#ab75766; 1:10,000
rabbit anti-human WDR77; EPR10708(B); Abcam; unconjugated Cat#ab154190; 1:2,000
rabbit anti-human PRMT5; EPR5772; Abcam; unconjugated Cat#ab109451; 1:10,000

Secondary Antibodies (Target; Clone; Brand; Fluorchorme and corresponding catalog numberr; dilution)
goat anti-rabbit IgG; polyconal; Promega; HRP Cat#W4011; 1:5,000
goat anti-mouse IgG; polyconal; Promega; HRP Cat#W4021; 1:5,000

Validation

All antibodies except for rabbit anti-human MAVS were validated by the supplier (Sigma-Aldrich, Cell Signaling, Invitrogen, Jackson ImmunoResearch, Millipore, Proteintech, Santa Cruz, Abcam, Promega) and were checked in our lab by comparing with the manufacturers or in-house results, using isotype controls.
Antibody against human MAVS was raised by immunizing rabbits with recombinant protein His-sumo-hMAVS-(aa-301-460), and it was previously used (Qi et al, 2017 Nature Communication) and further validated in this study (Fig. 1a, Fig. 2c, Fig. 4b-f, h, Fig. 5a, b, Supplementary Fig. 3a, Supplementary Fig. 4c-f, Supplementary Fig. 5b).
Flag (M2, Sigma-Aldrich, F3165): Anti Flag M2 antibody is used for the detection of Flag-tagged proteins. This monoclonal antibody is produced in mice and recognizes the FLAG sequence at the N-terminus, Met N-terminus, and C-terminus. Application: WB, IP, IHC, IF, Flow Cytometry. The antibody had a validation statement provided on the website of the manufacturer: https://www.sigmaaldrich.cn/CN/en/product/sigma/f3165.
Flag (Millipore, F7425): The rabbit Anti-FLAG polyclonal affinity antibody ANTI-FLAG recognizes the FLAG epitope located on FLAG fusion proteins. This antibody reacts with N-terminal, N-terminal-Met, and C-terminal FLAG fusion proteins. Application: dot blot, immunoblotting, immunoprecipitation, and immunocytochemistry assays. The antibody had a validation statement provided on the website of the manufacturer: https://www.sigmaaldrich.cn/CN/en/product/sigma/f7425.
HA (C29F4, Cell Signaling, 3724): HA-Tag (C29F4) Rabbit mAb detects exogenously expressed proteins containing the HA epitope tag. The antibody may cross-react with a protein of unknown origin ~100kDa. Species Reactivity: All Species Expected. Application: WB, IP, IHC, IF, Flow Cytometry. The antibody had a validation statement provided on the website of the manufacturer: https://www.cellsignal.com/products/primary-antibodies/ha-tag-c29f4-rabbit-mab/3724.
Tubulin (B-5-1-2, Sigma-Aldrich, T5168): Monoclonal Anti-α-Tubulin (mouse IgG1 isotype) is derived from the B-5-1-2 hybridoma produced by the fusion of mouse myeloma cells and splenocytes from an immunized mouse. Specificity: Recognizes an epitope located at the C-terminal end of the α-tubulin isoform in a variety of organisms. Application: WB, IF. The antibody had a validation statement provided on the website of the manufacturer: https://www.sigmaaldrich.cn/CN/en/product/sigma/t5168.
TBK1 (D1B4, Cell Signaling, 3504): Specificity: TBK1/NAK (D1B4) Rabbit mAb detects endogenous levels of total TBK1/NAK protein. Species Reactivity: Human, Mouse, Rat, Monkey. Application: WB, IP. The antibody had a validation statement provided on the

website of the manufacturer: https://www.cellsignal.com/products/primary-antibodies/tbk1-nak-d1b4-rabbit-mab/3504.
Phospho-TBK1/NAK (Ser172) (D52C2, Cell Signaling, 5483): Specificity: This Rabbit mAb detects endogenous levels of TBK1 only when phosphorylated at Ser172. This antibody may cross-react with phospho-IKKε. Species Reactivity: Human, Mouse. Application: WB, IP, IF, Flow Cytometry. The antibody had a validation statement provided on the website of the manufacturer: https://www.cellsignal.com/products/primary-antibodies/phospho-tbk1-nak-ser172-d52c2-xp-rabbit-mab/5483.
RIG-I (D14G6, Cell Signaling, 3743): Specificity: Rig-I (D14G6) Rabbit mAb detects endogenous levels of total Rig-I protein. Species Reactivity: Human, Mouse, Rat, Hamster, Monkey. Application: WB, IP. The antibody had a validation statement provided on the website of the manufacturer: https://www.cellsignal.com/products/primary-antibodies/rig-i-d14g6-rabbit-mab/3743.
Phospho-IRF-3 (Ser386) (E7J8G, Cell Signaling, 37829): Specificity: Phospho-IRF-3 (Ser386) (E7J8G) XP® recognizes endogenous levels of IRF-3 protein only when phosphorylated at Ser386. Species Reactivity: Human. Application: WB, IF, Flow Cytometry. The antibody had a validation statement provided on the website of the manufacturer: https://www.cellsignal.com/products/primary-antibodies/phospho-irf-3-ser386-e7j8g-xp-rabbit-mab/37829.
MAVS (Cell Signaling, 4983): Specificity: MAVS Antibody detects endogenous levels of total MAVS/VISA protein. The bands detected at 52 and 75 kDa correlate with those described by Seth et al. (2005). Species Reactivity: Mouse, Rat. Application: WB, IF, IP. The antibody had a validation statement provided on the website of the manufacturer: https://www.cellsignal.com/products/primary-antibodies/mavs-antibody-rodent-specific/4983.
IRF3 (Proteintech, 11312-1-AP): 11312-1-AP targets IRF3 in WB, RIP, IP, IHC, IF, FC, CoIP, ChIP, and ELISA applications and shows reactivity with human samples. The antibody had a validation statement provided on the website of the manufacturer: https://www.ptglab.com/products/IRF3-Antibody-11312-1-AP.htm.
TRAF3 (Santa Cruz, sc-1828): TRAF3 Antibody (H-122) is a rabbit polyclonal IgG; The antibody had a validation statement provided on the website of the manufacturer: https://www.scbt.com/p/traf3-antibody-h-122/?productCanUrl=traf3-antibody-h-122&_requestid=2260882.
Prohibitin (EP2803Y, Abcam, ab75766): Produced recombinantly (animal-free) for high batch-to-batch consistency and long-term security of supply. Rabbit monoclonal [EP2803Y] to Prohibitin. Suitable for: Flow Cyt (Intra), ICC/IF, WB, IP, IHC-P. Reacts with: Mouse, Rat, Human. The antibody had a validation statement provided on the website of the manufacturer: https://www.abcam.com/products/primary-antibodies/prohibitin-antibody-ep2803y-ab75766.html.
WDR77 (EPR10708(B), Abcam, ab154190): Key features and details
Produced recombinantly (animal-free) for high batch-to-batch consistency and long-term security of supply. Rabbit monoclonal [EPR10708(B)] to WDR77. Suitable for: Flow Cyt (Intra), WB, IHC-P, ICC/IF, IP. Reacts with: Mouse, Rat, Human. The antibody had a validation statement provided on the website of the manufacturer: https://www.abcam.com/products/primary-antibodies/wdr77-antibody-epr10708b-ab154190.html.
PRMT5 (EPR5772, Abcam, ab109451): Key features and details. Produced recombinantly (animal-free) for high batch-to-batch consistency and long-term security of supply. Rabbit monoclonal [EPR5772] to PRMT5. Suitable for: Flow Cyt (Intra), WB, IHC-P, ICC/IF, IP. Reacts with: Mouse, Rat, Human. The antibody had a validation statement provided on the website of the manufacturer: https://www.abcam.com/products/primary-antibodies/prmt5-antibody-epr5772-ab109451.html.

Eukaryotic cell lines

Policy information about cell lines and Sex and Gender in Research

Cell line source(s)	HEK293T was from American Type Culture Collection (ATCC). HEK293, HeLa, MEF and Vero cells were purchased from the Cell Resource Center (Shanghai Institute of Biochemistry and Cell Biology).
Authentication	Cell lines were authenticated by the companies we obtained them from. We checked the their morphology in culture and used low-passage cell cultures for all the experiments.
Mycoplasma contamination	Cell lines were routinely screened to avoid mycoplasma contamination. All cell lines tested negative for mycoplasma contamination.
Commonly misidentified lines (See ICLAC register)	No commonly misidentified cell line was used in the study.

Animals and other research organisms

Policy information about studies involving animals; ARRIVE guidelines recommended for reporting animal research, and Sex and Gender in Research

Laboratory animals	Wdr77fl/fl, Lyz2-Cre and Mavs knockout mice were in C57BL/6 background. Mice were maintained under a specific pathogen-free (SPF) condition. All mice were cultured in a suitable temperature and humidity environment (25 ℃, suitable humidity (typically 50%), 12-hour dark/light cycle), and fed with sufficient water and food. Eight to twelve weeks old mice were used for the experiments.
Wild animals	The study did not involve wild animals.
Reporting on sex	Sex was not considered in the study design.
Field-collected samples	The study did not involve samples collected in the field.
Ethics oversight	All animal experiments were performed in accordance with the guidelines of the Institutional Animal Care and Use Committee (IACUC) at the Shanghai Institute of Biochemistry and Cell Biology.

Note that full information on the approval of the study protocol must also be provided in the manuscript.
